# Identifying the vulnerable among the vulnerable: applying quantitative intersectionality methods to assess potential inequities in the HIV continuum of care for people living with schizophrenia in the united States

**DOI:** 10.1007/s00127-025-02972-7

**Published:** 2025-08-04

**Authors:** Paul Wesson, Eric Vittinghoff, Marilyn D. Thomas, Stephen Crystal, Richard Hermida, James Walkup, Francine Cournos, Mark Olfson, Christina Mangurian

**Affiliations:** 1 Department of Epidemiology and Biostatistics, School of MedicineUniversity of California, San Francisco UCSF, 550 16th Street, 3rd Floor, Box 0886, 94143, San Francisco, CA USA; 2 Department of Psychiatry and Behavioral Sciences, University of California, San Francisco, San Francisco, CA USA; 3 Institute for Health, Health Care Policy, and Aging Research, Rutgers University, New Brunswick, NJ USA; 4 Graduate School of Applied and Professional Psychology, Rutgers University, Piscataway, NJ USA; 5 Department of Psychiatry, Columbia University Vagelos College of Physicians and Surgeons, New York, NY USA; 6 Department of Epidemiology, Columbia University Mailman School of Public Health, New York, NY USA; 7 New York State Psychiatric Institute, New York, NY USA

**Keywords:** Intersectionality, Schizophrenia, HIV testing, Retention in HIV care, Medicaid, Mulit-level modeling

## Abstract

**Background:**

People living with schizophrenia face disproportionate risk of HIV, yet HIV testing remains low. Differential testing rates and engagement in care may be impacted by compounding social marginalization, partly linked to structural barriers. Grounded in intersectionality, we set out to identify the riskiest intersectional positions for HIV testing and engagement in HIV care in the United States.

**Methods:**

We created a retrospective cohort of people living with schizophrenia and matched controls, using 2012 national Medicaid claims data. We coded intersectional positions based on schizophrenia diagnosis, race/ethnicity, sex, and age. We used Multilevel Analysis of Individual Heterogeneity and Discriminatory Accuracy (MAIHDA) models to assess intersectional effects for two outcomes, HIV testing and retention in HIV care (RIC) defined as ≥2 CD4 or HIV viral load tests ≥90 days apart.

**Results:**

Of 777,887 patients in the testing cohort, 7.7% tested for HIV; 39% of the 17,913 patients in the RIC cohort were retained in care. In MAIHDA models without fixed effects, intersectional positions explained 12.7% of the variance in HIV testing and 7.4% of the variance in RIC. In final models including fixed and random effects, intersectional positions accounted for 1.4% of the variance in HIV testing and 0.8% of the variance in RIC. Older Black men with schizophrenia had lower-than-expected RIC prevalence in final models.

**Conclusion:**

Intersectional MAIHDA models can identify both vulnerable and resilient intersectional positions. The antagonistic intersectional effects for older Black men with schizophrenia highlight the need for targeted interventions to address structural barriers.

**Supplementary Information:**

The online version contains supplementary material available at 10.1007/s00127-025-02972-7.

## Introduction

People with severe mental illness (SMI) are a high-risk population for HIV and transmission. The nearly 10 million US adults living with SMI [1] have up to a 10-fold increased prevalence of HIV compared to the general population [2–5]. However, prevalence estimates for the SMI population range modestly across studies. A meta-analysis found a 6% prevalence of HIV among people with SMI in the US [2]. Another review found lower mean prevalence (1.8%), but included fewer studies, and many were self-report [3]. A multi-site cross-sectional study testing people in mental health settings estimated HIV prevalence in this population to be 4.8% [6]. Compared to the HIV prevalence among the general population of US adults, 0.5%, these studies indicate that people with SMI bear a disproportionate risk for HIV. Despite this elevated risk for HIV, HIV testing rates among the SMI population are insufficient relative to risk [7]. Testing gaps are likely driven by the multiple barriers faced by the SMI population to being tested, linked, and retained in HIV care [8, 9].

Socially marginalized populations often face the greatest HIV burden. Key populations at elevated risk for HIV include men who have sex with men (MSM), female sex workers (FSW), and people who inject drugs (PWID); the patterns of which populations are at greatest risk vary by country. In the US, the social patterning of HIV disparities is also well recognized according to racial, gender, income, and sexual orientation [10–17].

Compounding social marginalization could significantly impact HIV burden among the most vulnerable. The HIV care continuum refers to a model of care engagment detailing the proportion of people living with HIV (PLWH) who are: diagnosed and know their status, on antiretroviral medication, retained in care; and virally suppressed. Prior studies consistently show disparities in health and health care outcomes even within subgroups at elevated risk. For example, in the U.S. racial and ethnic minorities with SMI and diabetes are generally less likely to be treated than their White counterparts [18]. With respect to HIV and sexual orientation, half of U.S. Black MSM are projected to acquire HIV in their lifetime, compared to just 9% of White MSM [19]. This disparity exists despite evidence showing that Black MSM engage in fewer HIV-related risk behaviors than White MSM [20, 21].

Intersectionality, born out of Black feminist legal scholarship in the U.S, [22–24], is a normative framework for understanding differential health outcomes for people at the intersection of multiple categories of social identity and hierarchical positions of power (e.g., race, gender, sexual orientation, and socioeconomic status) [24–27]. Intersectionality focuses attention on the ways that experiences of those at different socio-demographic intersections are differentially shaped by social power in structural and interpersonal contexts [24, 28–31].

Outcomes along the HIV care continuum for the SMI population may reflect intersecting categories of social marginalization (e.g., race and sex). Given these dynamics, intersectionality is an effective theoretical framework and statistical method to identify subpopulations of people with SMI who are at especially high risk for HIV, and for disengaging from the care continuum. In identifying the riskiest intersectional positions, quantitative intersectionality methods can also inform effective and efficient interventions with a “matrix” (rather than “single axis”) approach. A single-axis intervention approach assumes all groups have equal benefit and does not consider heterogeneities. In contrast, a matrix approach, which identifies the most protective and riskiest intersectional positions, may be a more cost-effective intervention strategy.

Using data from a large, national, Medicaid sample, we set out to apply quantitative intersectionality to study inequities in HIV testing and care engagement, with a focus on people with diagnosed schizophrenia. This study was motivated by the overarching hypothesis that people diagnosed with schizophrenia, who are also members of other socially marginalized populations, will have poorer rates of engagement and retention in the HIV care continuum compared to people who belong to a single socially marginalized category, and beyond the cumulative effect of individual categories.

## Methods

### Overall study design

A retrospective cohort was created using national administrative Medicaid Analytic eXtract (MAX) data from the Centers for Medicare & Medicaid Services from January 1, 2012, to December 31, 2012. This time period corresponds to the fact that Medicaid data is often not made available until numerous years after it has been collected. Medicaid is a major payer of care for people living with schizophrenia and the primary payer for a significant portion of PLWH in the United States [32], covering approximately 36% of PLWH in 2012 [33]. This cohort has been described in detail previously [34].

### Study participants

From the larger Medicaid claims data, we constructed two cohorts for analysis: the “Testing” cohort (to analyze differences in HIV testing rates) and the retention in care (“RIC”) cohort (to analyze differences in retention in HIV care). Eligibility for the testing cohort included: (1) Medicaid beneficiaries aged 15–64 years as of December 31 during the observed calendar year, (2) at least 11 months of eligibility during the calendar year, and (3) residence in one of the 45 states with available Medicaid data during the study period. Individuals were excluded if there was an ICD-9 billing code for HIV infection (042, 079.53, V08, 795.71) at least 12 months before the 2012 calendar year. Eligibility for the RIC cohort included the same criteria as above for the testing cohort and that they were living with HIV as indicated by ICD-9 billing codes in the prior year. For both cohorts, people living with schizophrenia were identified by having at least one inpatient or at least two outpatient claims for schizophrenia (ICD-9 295.x; see Appendix Table 1 for variable definitions) within any 6-month period during the observed calendar year. An equal-sized frequency-matched control group without schizophrenia was also created for both cohorts. Controls were randomly sampled within strata defined by age (in decades), sex, race/ethnicity, and basis of Medicaid eligibility. For both cohorts, people with claims for bipolar disorder, psychosis not otherwise specified, delusional disorder, and pervasive developmental disorder were excluded from the controls. We further restricted the cohort to the most recent year of data, 2012.

**Table 1:** Demographic distribution of HIV testing and retention in care (RIC) cohorts from medicaid claims data, 2012

	HIV Testing cohort		RIC cohort	
	*n*	%	*n*	%
Total	777,887	100	17,913	100
**SMI**				
Schizophrenia	331,575	42.6	8,038	44.9
Control	446,312	57.4	9,875	55.1
**Sex**				
Female	366,598	47.1	8,135	45.4
Male	411,289	52.9	9,778	54.6
**Race/Ethnicity**				
NH White	359,285	46.2	3,199	17.9
NH Black	292,684	37.6	11,662	65.1
Hispanic	89,963	11.6	2,815	15.7
Asian and Pacific Islander	28,591	3.7	155	0.9
American Indian	7,364	1.0	82	0.5
**Age Group**				
15–29	160,798	20.7	1,267	7.1
30–49	338,828	43.6	9,049	50.5
50–64	278,261	35.8	7,597	42.4
Normal Risk Pregnancy	19,641	2.5	271	1.5
High Risk Pregnancy	6,877	0.9	146	0.8
HIV Test	59,522	7.7	NA	-
Retained in care	NA	-	7,047	39.3

RIC = Retention in Care; SMI = Severe Mental Illness; NH = non-Hispanic

Retained in care defined as two or more CD4 or HIV viral load tests at least 90 days apart from each other, within the same calendar year

### Measures

We constructed intersectional positions based on the cross-classification of patient sex (male, female), race/ethnicity (non-Hispanic White, non-Hispanic Black, Hispanic, Asian and Pacific Islander, Native American), age (15–29 years, 30–49 years, 50–64 years), and presence/absence of schizophrenia diagnosis. We examined two outcomes, separately: HIV testing (defined as at least one claim for an HIV test within the calendar year) and retention in HIV care (defined as at least two CD4 or HIV viral load tests at least 90 days apart within the same calendar year).

### Analysis

Due to the number of sociodemographic variables used to create intersectional positions, we chose the Multilevel Analysis of Individual Heterogeneity and Discriminatory Accuracy (MAIHDA) model to explore evidence for intersectional effects [30, 35]. The intersectional MAIHDA model is a multi-level modeling approach used to study intersectionality quantitatively. Drawing from social epidemiology and multi-level modeling in which observations within physical clusters (e.g., schools, neighborhoods) are correlated, intersectional MAIHDA models nest individuals (level 1 of the multi-level model) within clusters in social space defined by their intersectional position (level 2 of the multi-level model). For example, one intersectional position created was Black females 30–49 years without schizophrenia. All participants in the cohort with these characteristics were grouped into a single intersectional cluster. The multi-level model accounts for the assumed greater correlation of observations within the same clusters through the random effects, supporting valid estimation of between-group and within-group variation. There were 58 clusters in total, defined by unique combinations of sex, race/ethnicity, age, and schizophrenia diagnosis.

We used mixed effects logistic regression to model both of our binary outcomes (HIV testing and retention in HIV care). For each outcome, we built two models. The first model, Model 1, included only the intercept and the intersectional positions as random effects. Model 2 included the intercept, the random effects, and each of the variables defining the intersectional positions (sex, race/ethnicity, age, schizophrenia diagnosis) as individual fixed effects. The US Preventive Services Task Force recommends that clinicians screen for HIV in all pregnant persons [36]. Therefore, we also included claims for high-risk pregnancy and normal risk pregnancy as additional fixed effects in Model 2 to account for increased health care engagement among those receiving prenatal care.

For each model, we calculated the variance partition coefficient (VPC) and the area under the receiver operator curve (AUROC). The VPC, also known as the intraclass correlation, is a measure of the correlation of the outcome between individuals within intersectional strata (i.e., the percentage of the variability in the outcome that is attributed to the intersectional positions/random effects). The VPC is calculated as,

$$\:VPC=ICC=\frac{{\sigma\:}_{u}^{2}}{{\sigma\:}_{u}^{2}+{\sigma\:}_{e}^{2}}$$

where ($\:{\sigma\:}_{u}^{2}$) is the between-strata (intersectional position) variance, and ($\:{\sigma\:}_{u}^{2}+{\sigma\:}_{e}^{2}$) is the total variance. The AUROC is a measure of the discriminatory accuracy of the model and can be interpreted as the probability that the model can correctly classify an individual observation as having the outcome or not. Typically, an AUROC ≥0.70 is considered a well-fitting model. For Model 2, we also calculated the proportional change in variance (PCV), which is the proportion of the total variance from Model 1 that has been explained by adding the fixed effects from Model 2. The PCV is calculated as,

$$\:PCV=\frac{{\sigma\:}_{u\left(null\:model\right)}^{2}-{\sigma\:}_{u\left(main\:effects\:model\right)}^{2}}{{\sigma\:}_{u\left(null\:model\right)}^{2}}$$

where $\:{\sigma\:}_{\left(null\:model\right)}^{2}$ is the between-stratum variance for the null model (random effects only) and $\:{\sigma\:}_{\left(main\:effects\:model\right)}^{2}$ is the between-stratum variance for the main effects model (random effects and fixed effects). For both outcomes, we used Model 2 to calculate the predicted mean prevalence of the outcome for each intersectional position and plotted the results to map the distribution of risk across all groups.

To assess intersectional effects, we calculated the attributable risk due to interaction (ARI) as the difference between the total predicted values for each stratum (i.e., intersectional position) and the stratum-level predictions based on the additive main effects alone. This is equivalent to the stratum residuals featured in other MAIHDA studies [37, 38]. After calculating 95% confidence intervals (CIs) on the ARI, if the 95% CI did not include the null (0), then the predicted mean prevalence for the intersectional position was determined to be greater than (ARI = positive) or less than (ARI = negative) the mean prevalence that would be expected based on the additive combination of single-axis covariates. Put another way, ARI gives the increased or reduced prevalence that goes beyond simply adding together the independent main or fixed effects of each social position. To map the distribution of risk across all 58 intersectional positions, we plotted the predicted mean prevalence and confidence intervals for each intersectional position separately for each outcome. We color-coded the predicted mean prevalence according to the ARI to note synergistic intersectional effects (positive ARI), antagonistic intersectional effects (negative ARI), or as expected based on the additive combination of fixed effects.

As a sensitivity analysis, we re-ran the MAIHDA models without the high-risk pregnancy and normal risk pregnancy as fixed effects. Because nonpsychiatric outpatient medical visits can also increase the odds of HIV testing for people with schizophrenia [39], we ran additional sensitivity analyses controlling for substance use disorders and sexually transmitted infections in MAIHDA models.

### Ethics

This study was reviewed by the UCSF institutional review board (#17-21998) and deemed exempt because data were de-identified.

## Results

The HIV testing cohort included 777,887 patients in 2012, while 17,913 patients were included in the RIC cohort (Table 1). There was a similar proportion of patients with a claim having a diagnosis for schizophrenia in both cohorts (43% in the HIV testing cohort, 45% in the retention in care cohort). Most patients in both cohorts were male (~ 54%). While a plurality of the HIV testing cohort was non-Hispanic White (46.2%), most patients in the RIC cohort were non-Hispanic Black (65.1%). Below, we describe the modeling results for each cohort.

### Testing cohort

**Fig. 1:**
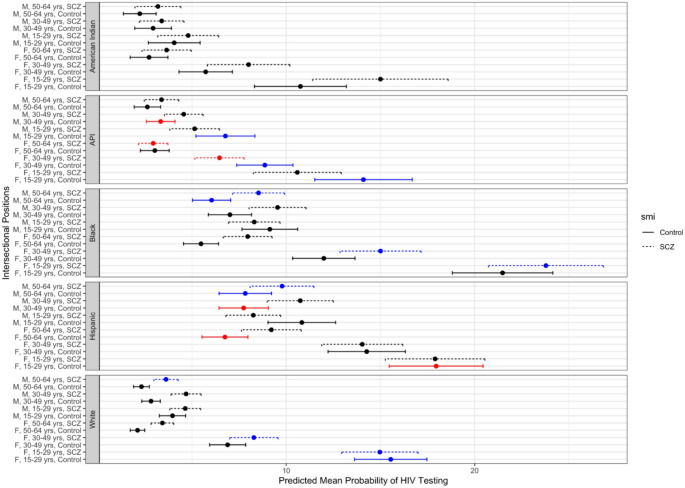
Predicted mean probability of HIV testing in 2012 Medicaid population by intersectional positions (race/ethnicity, sex, age, mental illness). Black dot and error bar indicates that predicted mean prevalence is as expected based on additive combination of fixed effects. Red dot and error bar indicates that predicted mean prevalence is less than expected based on additive combination of fixed effects (antagonistic interaction). Blue dot and error bar indicates that predicted mean prevalence is more than expected based on additive combination of fixed effects (synergistic interaction)

In 2012, 7.65% of the HIV testing cohort had a claim for an HIV test. By intersectional position (cross-categorizations of schizophrenia diagnosis, sex, race/ethnicity, and age group), the prevalence of HIV claims ranged from 2.11% among White females aged 50–64 without schizophrenia to 22.14% among Black females ages 15–30 with schizophrenia (Fig. 1).

The results from adjusted multivariate analysis (Table 2) indicated that people living with schizophrenia were more likely to receive an HIV test compared to people without schizophrenia (aOR = 1.27; 95% CI: 1.13, 1.42) (Table 2). Compared to females, males were less likely to receive an HIV test (aOR = 0.73; 95% CI: 0.65, 0.83). Both Black (aOR = 2.18; 95% CI: 1.84, 2.61) and Hispanic (aOR = 2.36; 95% CI: 1.99, 2.83) individuals were more likely to receive an HIV test compared to White individuals. There was an inverse dose-response relationship with age, with older age groups less likely to test for HIV.

**Table 2:** Multilevel analysis of individual heterogeneity and discriminatory accuracy (MAIHDA) analysis of HIV testing in medicaid data, 2012

Measure of Association	Model 1 (RE only)	Model 2 (RE + FE)
SMI		
No SMI		***Reference***
SMI		1.27 (1.13, 1.42)
Sex		
Female		***Reference***
Male		0.73 (0.65, 0.83)
Race		
White		***Reference***
Black		2.18 (1.84, 2.61)
Hispanic		2.36 (1.99, 2.83)
API		1.07 (0.89, 1.28)
American Indian		0.91 (0.74, 1.13)
Age Group		
15–29		***Reference***
30–49		0.79 (0.68, 0.90)
50–64		0.52 (0.45, 0.60)
High Risk Pregnancy		1.99 (1.88, 2.12)
Normal Risk Pregnancy		7.32 (7.10, 7.61)
Measures of variance and discriminatory accuracy		
Variance	0.691	0.217
VPC	0.127	0.014
PCV		0.685
AUROC	0.681	0.704

RE = Random Effects; FE = Fixed Effects; SMI = Severe Mental Illness; API = Asian and Pacific Islander; VPC = Variance Partition Coefficient; PCV = Proportional Change in Variance; AUROC = Area Under the Receiver Operator Curve

The results of the MAIHDA model indicate that, overall, modeling the intersectional positions explained 12.7% of the variance in HIV testing (Table 2). When fixed effects were added to the model, this VPC was reduced to 1.4%. Adding fixed effects to the model modestly improved the AUROC from 0.681 to 0.704, and decreased the variance from 0.69 to 0.22.

Figure 1 maps the distribution of risk (i.e., the predicted mean prevalence) for HIV testing across all intersectional positions. Figure 1 also identifies groups suggestive of intersectional effects; that is, the predicted mean prevalence is greater than or less than the predicted mean prevalence that would be expected from the additive combination of fixed effects. The three intersectional positions with the lowest predicted probability of HIV testing were White females ages 50–64 without SMI (predicted mean prevalence = 2.11%), American Indian males ages 50–64 without SMI (predicted mean prevalence = 2.22%), and White males ages 50–64 without SMI (predicted mean prevalence = 2.31%). In contrast, the three intersectional positions with the highest predicted probability of HIV testing were Hispanic females ages 15–29 without SMI (predicted mean prevalence = 17.96%), Black females ages 15–29 without SMI (predicted mean prevalence = 21.48%), and Black females ages 15–29 with SMI (predicted mean prevalence = 23.79%). Thirteen of the 58 intersectional positions had greater than expected predicted mean prevalence of HIV testing (22.4%). Six of the nearly sixty intersectional positions (10.3%) had less than expected predicted mean prevalence of HIV testing.

### RIC cohort

**Fig. 2:**
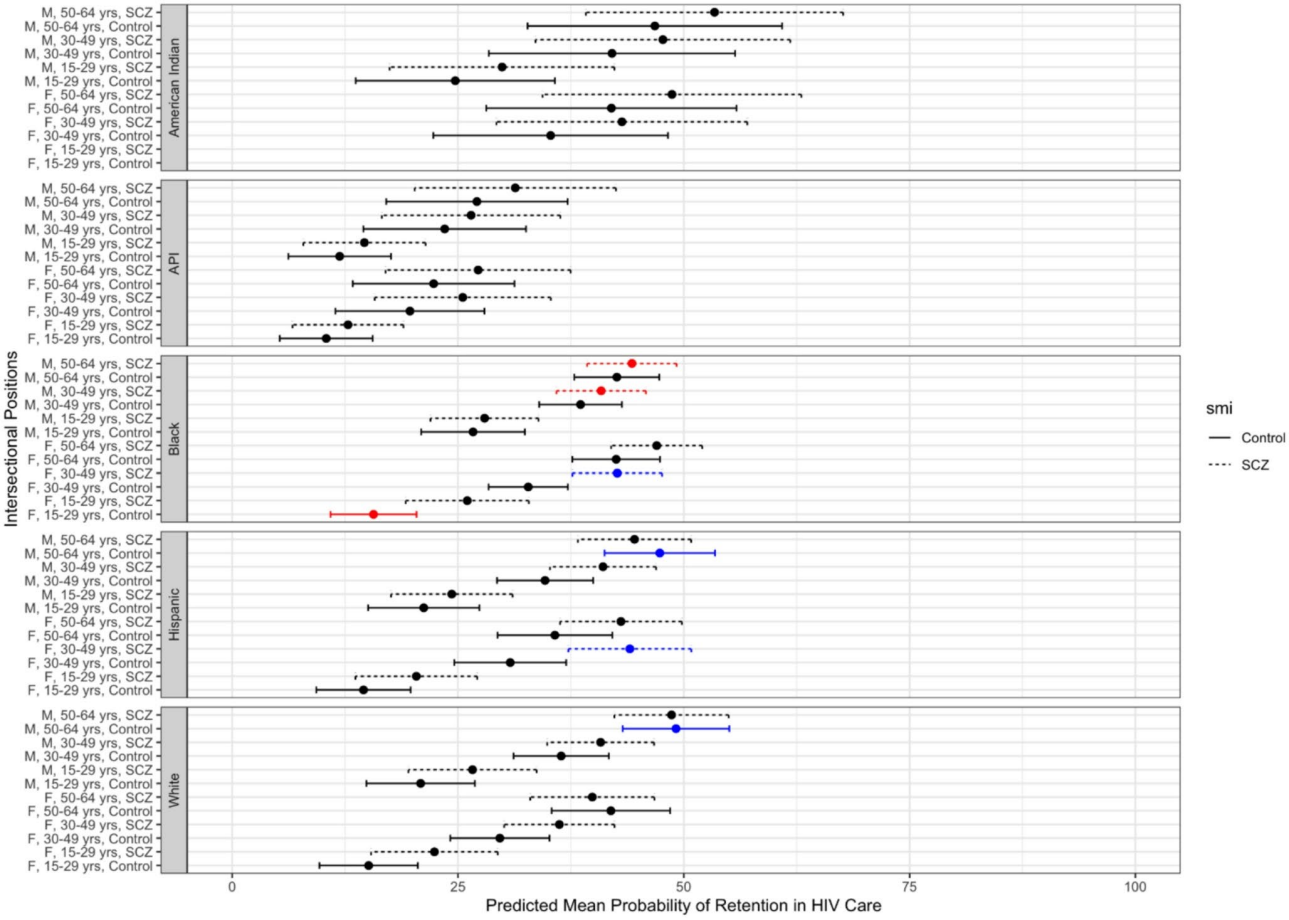
Predicted mean probability of retention in HIV care in 2012 Medicaid population by intersectional positions (race/ethnicity, sex, age, mental illness). Black dot and error bar indicates that predicted mean prevalence is as expected based on additive combination of fixed effects. Red dot and error bar indicates that predicted mean prevalence is less than expected based on additive combination of fixed effects (antagonistic interaction). Blue dot and error bar indicates that predicted mean prevalence is more than expected based on additive combination of fixed effects (synergistic interaction)

Among those with an HIV diagnosis, 39.3% were retained in care in 2012. By intersectional position, retention in care ranged from 10.4% among Asian and Pacific Islander females ages 15–29 without schizophrenia to 53.4% among American Indian males ages 50–64 with schizophrenia (Fig. 2).

Similar to HIV testing, people living with schizophrenia were more likely to be retained in care compared to people without schizophrenia (aOR = 1.27; 95% CI: 1.11, 1.46) (Table 3). Several other associations, however, were reversed. Regardless of schizophrenia claims, females were less likely to be retained in HIV care compared to males (aOR = 0.84; 95% CI: 0.73, 0.96). There were no racial/ethnic differences in retention in care, with the exception of Asians and Pacific Islanders, who, compared to White patients, were less likely to be retained in care (aOR = 0.50; 95% CI: 0.33, 0.76). Older age was associated with greater retention in HIV care.

**Table 3:** Multilevel analysis of individual heterogeneity and discriminatory accuracy (MAIHDA) analysis of retention in HIV care (≥2 CD4 or viral load tests ≥90 days apart) in medicaid data, 2012

Measure of Association	Model 1 (RE only)	Model 2 (RE + FE)
SMI		
No SMI		***Reference***
SMI		1.27 (1.11, 1.46)
Sex		
Female		0.84 (0.73, 0.96)
Male		***Reference***
Race		
White		***Reference***
Black		1.08 (0.91, 1.28)
Hispanic		0.97 (0.81, 1.17)
API		0.50 (0.33, 0.76)
American Indian		1.21 (0.76, 1.95)
Age Group		
15–29		***Reference***
30–49		2.12 (1.72, 2.61)
50–64		2.69 (2.18, 3.32)
High Risk Pregnancy		1.82 (1.13, 2.97)
Normal Risk Pregnancy		0.44 (0.29, 0.65)
Measures of variance and discriminatory accuracy		
Variance	0.513	0.163
VPC	0.074	0.008
PCV	-	0.681
AUROC	0.579	0.579

The intersectional positions alone explained 7.4% of the variance in retention in care in 2012 (Table 3). This was reduced to less than 1% when fixed effects were added to the model. Adding fixed effects to the MAIHDA model for retention in care did not improve the AUROC, which fell below the 0.7 threshold for an acceptable fit.

Figure 2 maps the distribution of the predicted mean probability of retention in HIV care in 2012. The predicted mean probability of retention in HIV care ranged from 10.41 to 53.4%. The three intersectional positions with the lowest predicted mean prevalence of retention in HIV care were: API females ages 15–29 without SMI (predicted mean = 10.41%), API males ages 15–29 without SMI (predicted mean = 11.89%), and API females ages 15–29 with SMI (predicted mean = 12.89%). The three intersectional positions with the highest predicted mean prevalence of retention in HIV care were: American Indian females ages 50–64 with SMI (predicted mean = 48.69%), White males ages 50–64 without SMI (predicted mean = 49.14%), and American Indian males ages 50–64 with SMI (predicted mean = 53.4%). Three intersectional positions had lower than expected predicted mean prevalence of retention in HIV care: Black males ages 50–64 with SMI, Black males ages 30–49 with SMI, and Black females ages 15–29 without SMI. Four intersectional positions had higher than expected predicted mean prevalence of retention in HIV care: Black females ages 30–49 with SMI, Hispanic males ages 50–64 without SMI, Hispanic females ages 30–49 with SMI, and White males ages 50–64 without SMI. Other key findings by intersectional position are highlighted in Table 4.

**Table 4:** Key findings by outcome and intersectional position

HIV Testing	Retention in HIV Care
Young Black women with a schizophrenia diagnosis were most likely to have a claim for an HIV test (greater than their predicted probability)	Older Black men with a schizophrenia diagnosis were less likely to be retained in care (compared to their predicted probability)
Middle-aged API men and older API women with a schizophrenia diagnosis were among the least likely to have a claim for an HIV test (lower than the expectation from their predicted probability)	Younger Black women without a schizophrenia diagnosis were among the least retained in care (lower than the expectation from their predicted probability)
Across intersectional positions, American Indian men and women were among the least likely to have a claim for an HIV test (consistent with their predicted probabilities)	Older White men without a schizophrenia diagnosis were among the most likely to be retained in care (greater than their predicted probability)

### Pregnancy analysis

The MAIHDA models with and without the pregnancy claims as confounders differed with respect to the independent associations of sex and age with HIV testing (Appendix Table 2). In models without the pregnancy claims confounders, males were even less likely compared to females to have a claim for HIV testing (aOR = 0.57; 95% CI: 0.49, 0.67). This was also the trend for older patients compared to those in the 15–29 years age group. Including the pregnancy claims confounders accounted for an additional 12% of the variance in the data explained by the model. The two models for retention in HIV care, with and without pregnancy claims confounders, were largely similar with respect to estimated fixed effects and measures of variance and discriminatory accuracy (Appendix Table 3).

When we included substance use disorder and STIs as additional fixed effects in sensitivity analyses, schizophrenia was no longer associated with HIV testing (Appendix Table 4). While the model fit improved marginally, most other parameters and predicted probabilities for intersectional positions remained largely unchanged (Appendix Table 4, Appendix Fig. 3). There were no differences in model estimates for the retention in care outcome models (Appendix Table 5, Appendix Fig. 4).

## Discussion

In this study, we used intersectional MAIHDA models to assess whether a schizophrenia diagnosis, along with compounding and interacting axes of social marginalization, was associated with lower HIV testing and reduced engagement in the HIV continuum of care. Prior to adding fixed effects to the model, nearly 13% of the variability in HIV testing could be attributed to intersectional strata based on schizophrenia diagnosis, race/ethnicity, sex, and age. Even after adding fixed effects to the model, 1.4% of the variability in HIV testing could still be attributed to intersectional position, highlighting the persistent influence of social positioning on HIV testing behavior. Further analysis detected intersectional effects for 19 intersectional positions; that is, the predicted mean prevalence was greater or less than the expected mean prevalence based on additive combination of covariates. In contrast, our analysis of inequities in retention in care found little evidence for intersectional effects, as less than 1% of the variability in retention could be attributed to intersectional positions.

Contrary to our hypothesis, we did not find that a schizophrenia diagnosis, or most intersectional positions that included a schizophrenia diagnosis, was associated with lower HIV testing and retention in care. In fact, MAIHDA models with fixed effects revealed that a schizophrenia diagnosis was associated with a greater likelihood of both having an HIV test within the past year and being retained in HIV care, a finding that has been reported in some earlier studies [34, 39, 40]. A closer examination of intersectional positions, however, point to potential gaps in care for older Black men with schizophrenia, who were retained in care less than expected.

The results indicating lower than expected retention in HIV care among older Black men with schizophrenia warrants further investigation. Little exists in the literature on the unique challenges experienced by this specific intersectional position. However, previous studies have identified low rates of engagement in HIV care among Black men who have sex with men (BMSM) as well as low rates of engagement in psychiatric care among Black men with schizophrenia [41, 42]. In a qualitative study evaluating the experience of BMSM clients with integrated behavioral and clinical health care services at eight demonstration sites across the United States, Daniels et al. identified various barriers to optimal engagement in HIV care among BMSM [42]. Perceived stigma and discrimination emerged as a prevalent theme for care disengagement, with clients noting that they were being perceived “differently” due to their identities (racial identity, sexual orientation, and HIV status). This was connected to experiences of judgement, inadequate provider sensitivity, and breaches of confidentiality, all of which damaged the patient-provider relationship. These experiences of intersectional stigma could be compounded with a schizophrenia diagnosis and make managing them more challenging. Wagstaff et al. note high rates of HIV care disengagement and disengagement from adult psychiatric outpatient services across multiple countries [43]. In their qualitative study of Black male patients with schizophrenia in the UK with a history of disengagement from mental health services, the authors note the stigma of being associated with mental health services as having a detrimental effect on their relationship with their community. Participants also felt that mental health services were “coercive,” “hounding” them about their medication. In addition to intersectional stigma, compounding structural barriers such as lack of transportation and inflexible or inconvenient work schedules likely also impact lower engagement in HIV care for older Black men with schizophrenia. Daniels et al. also noted that BMSM clients highlighted telehealth appointments as a convenient option to overcome such barriers and as less alienating with respect to the intersectional stigma they experienced during in-person medical visits. The recent expansion of telehealth visits in response to the COVID-19 pandemic should be studied for its potential impact on narrowing the retention in HIV care gap for this intersectional position. Separately, the growing body of evidence showing that Black men are misdiagnosed with schizophrenia at higher rates compared to other groups might also contribute to our findings [44–46]. The high rates of false diagnoses of schizophrenia among Black men signals racial discrimination in the healthcare system and can result in medical mistrust among patients. While we cannot, with this study, empirically speak to the attributable risk of medical mistrust on HIV care disengagement for older Black men with schizophrenia, further investigation is warranted.

Mapping the distribution of risk between groups and outcomes facilitated interesting contrasts that might otherwise go unnoticed using a “single axis” approach. For example, young (ages 15–29 years) Black females with schizophrenia had the highest predicted mean prevalence of HIV testing among all the intersectional positions. Their predicted mean prevalence also showed evidence for synergistic intersectional effects (i.e., the predicted mean prevalence was greater than expected based on additivity). In contrast, White males without schizophrenia (across all age groups) had among the lowest predicted mean prevalence of HIV testing. These differences may speak to differences in risk perception by individuals and their care providers. However, when exploring inequities in retention in care, we found that older White males without schizophrenia, a group that likely experiences the least marginalization and structural oppression, were among the most likely to be retained in care and had a higher-than-expected predicted mean prevalence. Younger Black females without schizophrenia, on the other hand, were among the least retained in care and had a lower-than-expected predicted mean prevalence. This could signal reduced access to health care services or other barriers to care that must be removed.

Our multivariable regression modeling (Model 2) reveals several notable trends: Medicaid beneficiaries with SMI (compared to the control group) were more likely to have an HIV test and to be retained in HIV care; females (compared to males) were more likely to test for HIV, but less likely to be retained in HIV care; and there was a dose-response relationship with respect to age, where older individuals were less likely to get an HIV test but more likely to be retained in HIV care. Black and Hispanic individuals were more likely (compared to White individuals) to test for HIV, but no differences were observed for retention in HIV care. These trends are consistent with the past 12-month HIV testing results from the 2012 Behavioral Risk Factor Surveillance System survey [47]. The intersectional lens provided by MAIHDA models lends further nuance to this examination of heterogeneity of vulnerability. For example, while younger people were more likely to have an HIV test, this trend was most pronounced among females. Across racial and ethnic groups, the predicted prevalence of HIV testing was largely similar for males regardless of age.

The intersectional MAIHDA approach is a useful tool for identifying priority populations and addressing HIV and related disparities in the US. Our results also highlight the low testing among both American Indians and API groups (across intersectional positions)-- groups often relegated to the racial category of “Other.” Our models specifically call attention to the low prevalence of testing among men in these groups as well as older women. Among API, specifically, middle-aged men and older women with schizophrenia not only had among the lowest HIV testing prevalence, but testing among these groups was also lower than expected. Further investigation is warranted to understand if this low prevalence of HIV testing is due to structural barriers or low risk perception.

Our findings should be understood in the context of several important limitations. First, our analysis is based on Medicaid claims data. Billing data are used as a proxy for what occurred during the medical visit but does not reveal what is in the actual medical record. Especially with respect to retention in HIV care, we are limited in not having viral load data, which is the most accurate assessment of a person’s HIV care. Services that are not billed, such as HIV testing in community settings, are not captured in this dataset. Second, our use of the CDC’s definition of retention in care, and restricting our analysis to the 2012 calendar year, risks under-counting the number of people retained in care if they received at least two care visits at least three months apart within twelve consecutive months, but those months straddled two calendar years (e.g., 2011 and 2012, or 2012 and 2013). Third, claims data do not include measures of sexual orientation, gender identity, or substance use, which are all highly relevant in the epidemiology of HIV and be additional cofounders impacting access/engagement. Fourth, due to delays in Medicaid data availability, we were constrained to use data from 2012 as the most recent available data. However, given that our study is framed around intersecting structural inequities, the testing and care dynamics observed likely persist well after this data was collected. Recent developments in MAIHDA models propose expanding its functionality to include random slopes which, in our case, could allow for the investigation of differences in trends over time by intersectional position (i.e., difference in differences) [48]. Given wide variation in state-level HIV testing among this population across states [34], another proposed development is the inclusion of random coefficients, which, in our case, could be applied to assess differences in intersectional positions by geography. The latter may be useful in further understanding the impact of context and structural disadvantage when studying disparities in the HIV care continuum, especially with respect to states that did not expand Medicaid coverage. For example, non-expansion states may only cover “medically necessary” HIV testing, wheras states with expanded Medicaid coverage will also include “routine” HIV screening (i.e. HIV testing offered to everyone between the ages of 13 and 64, regardless off perceived risk) [49]. Lastly, the use of Medicaid claims data means that our results cannot be generalized to the broader US population, including uninsured or commercially insured individuals. Relatedly, services not billed to Medicaid, such as HIV testing in community settings, is not captured in our data. Our inability to examine these services could result in an underestimation of HIV testing for some intersectional positions, particularly those more likely to be uninsured. However, our results are relevant to the broad population of people with SMI because an estimated 67% of people with diagnosed schizophrenia access medical care through Medicaid and speaks to a population with greater unmet needs given the relationship between Medicaid coverage, income, and disability [50].

The intersectional MAIHDA approach is a useful tool for identifying priority populations for federal efforts to address the HIV epidemic and the hardest hit communities [51]. HIV testing and care interventions often take a “single axis” approach; that is, targeting outreach efforts towards single identities (racial, sexuality, gender identity, mental health status). While our models show that these variables were independently associated with HIV care outcomes (when included in the model as fixed effects), the results also indicate that there was remaining variability explained by the random effects and, therefore, an intersectional (“matrix”) approach may also be warranted. Specifically, our intersectional MAIHDA models identified older Black men with schizophrenia as an especially vulnerable group with lower-than-expected retention in HIV care. This could be the result of compounding structural barriers to care as well as perceptions of intersectional stigma. This should be investigated further, along with testing interventions such as telehealth, to close the gap in the HIV care continuum.

## Supplementary Information

Below is the link to the electronic supplementary material.

Supplementary Material 1

## Data Availability

Data is provided within the manuscript or supplementary information files.
