# Supplementary material for: Identifying the vulnerable among the vulnerable: applying quantitative intersectionality methods to assess potential inequities in the HIV continuum of care for people living with schizophrenia in the united States
Source: Soc Psychiatry Psychiatr Epidemiol. 2025 Aug 4;61(4):603–14. doi: 10.1007/s00127-025-02972-7 (PMC13021718; doi:10.1007/s00127-025-02972-7)
Supplement: Supplementary file 1 — Supplementary Material 1 [file 127_2025_2972_MOESM1_ESM.docx]

**Appendix Table 1. ICD-9 diagnosis codes and CPT codes**

| Variable | ICD-9 Code | CPT code | Definition |
| --- | --- | --- | --- |
| HIV Testing |  | 86311 | HIV antigen testing 13 |
|  |  | 86312 | HIV/AIDS testing, but it is specifically “HTLV-III antibody detection, ELISA” 13 |
|  |  | 86314 | HIV/AIDS testing, but it is specifically “HTLV-III antibody detection” 13 |
|  |  | 86689 | Western Blot for HIV antibody 13-18 |
|  |  | 86701 | (HIV-1) |
|  |  | 86702 | (HIV-2) |
|  |  | 86703 | HIV Antibodies, HIV-1/HIV-2, EIA with Reflex to HIV-1, Western Blot |
|  |  | 87390 | HIV-1 direct AG (ICD), ELISA |
|  |  | 87391 | HIV-2 antigen |
|  |  | 87389 | HIV-1 antigen([s], with HIV-1 and HIV-2 antibodies, single result) |
|  |  | 87534 | HIV-1 Nucleic Acid Detection – Direct Probe Technique |
|  |  | 87535 | HIV-1 Nucleic Acid Detection – Amplified Probe Technique |
|  |  | 87536 | HIV-2 Nucleic Acid Quantification13-17 |
|  |  | 87537 | HIV-2 Nucleic Acid Detection – Direct Probe Technique |
|  |  | 87538 | HIV-2 Nucleic Acid Detection – Amplified Probe Technique |
|  |  | 87539 | HIV-2 Nucleic Acid Quantification |
| Retention in (HIV) Care |  | 87536 | HIV-1 Nucleic Acid Quantification13-17 |
|  |  | 86360 | T cells; absolute CD4 count WITH ratio |
|  |  | 86361 | T cells; absolute CD4 count |
| Normal Risk Pregnancy | V22 |  | Normal pregnancy |
|  | V22.0 |  | Supervision of normal first pregnancy |
|  | V22.1 |  | Supervision of other normal pregnancy |
|  | V24.0 |  | Postpartum care and examination immediately after delivery |
|  | V22.2 |  | Pregnant state, incidental |
| High Risk Pregnancy | V23 |  | High risk pregnancy |
|  | V23.0 |  | Supervision of high-risk pregnancy with history of infertility |
|  | V23.1 |  | Supervision of high-risk pregnancy with history of trophoblastic disease |
|  | V23.2 |  | Supervision of high-risk pregnancy with history of abortion |
|  | V23.3 |  | Supervision of high-risk pregnancy with grand multiparity |
|  | V23.4 |  | Supervision of high-risk pregnancy with other poor obstetric history |
|  | V23.5 |  | Supervision of high-risk pregnancy with other poor reproductive history |
|  | V23.7 |  | Supervision of high-risk pregnancy with insufficient prenatal care |
|  | V23.8 |  | Supervision of other high-risk pregnancy |
|  | V23.9 |  | Supervision of unspecified high-risk pregnancy |
| Severe Mental Illness (SMI)/Schizophrenia | 295 (with wildcard) |  | Schizophrenic Disorders |
|  | 295.7 (with wildcard) |  | Schizoaffective disorder |
|  | 295.4 |  | Schizophreniform disorder |
| Substance Use Disorder |  |  |  |
| *Opioid Use Disorder* | 304.0 (with wildcard) |  | Opioid type dependence |
|  | 305.5 (with wildcard) |  | Nondependent opioid abuse |
| *Cocaine use Disorder* | 304.2 (with wildcard) |  | Cocaine dependence |
|  | 305.6 (with wildcard) |  | Nondependent cocaine abuse |
| *Other Drug Use Disorder* | 304.6 (with wildcard) |  | Other, specified drug dependence |
|  | 304.8 (with wildcard) |  | Combinations excluding opioids |
|  | 304.9 (with wildcard) |  | Unspecified drug dependence |
|  | 305.9 (with wildcard) |  | Other, mixed or unspecified drug abuse |
|  | 648.3 (with wildcard) |  | Drug dependence complicating pregnancy, childbirth, or the puerperium |
| *Substance Use Disorder* | 304.0 (with wildcard) |  | Drug dependence on morphine type drugs |
|  | 304.1 (with wildcard) |  | Sedative, hypnotic or anxiolytic dependence |
|  | 304.2 (with wildcard) |  | Drug dependence on cocaine |
|  | 304.4 (with wildcard) |  | Drug dependence on amphetamine type drugs and other psychostimulants |
|  | 304.5 (with wildcard) |  | Hallucinogen dependence |
|  | 304.6 (with wildcard) |  | Drug dependence on other drug |
|  | 304.7 (with wildcard) |  | Drug dependence on combinations of morphine type dugs with any other drug |
|  | 304.8 (with wildcard) |  | Drug dependence on combinations excluding morphine type drug |
|  | 304.9 (with wildcard) |  | Drug dependence on unspecified drug |
|  | 305.4 (with wildcard) |  | Sedative, hypnotic, or anxiolytic abuse – unspecified |
|  | 305.5 (with wildcard) |  | Opioid abuse – unspecified |
|  | 305.6 (with wildcard) |  | Cocaine abuse - unspecified |
|  | 305.7 (with wildcard) |  | Amphetamine or related acting sympathomimetic abuse – unspecified |
|  | 305.9 (with wildcard) |  | Nondependent abuse- other, mixed or unspecified |
|  | 648.3 (with wildcard) |  | Drug dependence of mother, antepartum and postpartum condition or complication |
|  | 769.0 (with wildcard) |  | Nonspecific abnormal toxicological findings |
| Sexually Transmitted Infections (STIs) |  |  |  |
| *Herpes Simplex* | 054 (with wildcard) |  | Herpes Simplex |
| *Chlamydia* | 77.98 |  | Unspecified diseases of conjunctiva due to chlamydiae |
|  | 78.88 |  | Other specified diseases due to chlamydiae |
|  | 79.98 |  | Unspecified chlamydial infection |
|  | 99.41 |  | Other nongonococcal urethritis, chlamydia trachomatis |
|  | 099.54 (with wildcard) |  | Other veneral diseases due to chlamydia trachomatis |
| *Syphilis* | 091 (with wildcard) |  | Early syphilis symptomatic |
|  | 092 (with wildcard) |  | Early syphilis latent |
|  | 093 (with wildcard) |  | Cardiovascular syphilis |
|  | 094 (with wildcard) |  | Neurosyphilis |
|  | 095 (with wildcard) |  | Other forms of late syphilis with symptoms |
|  | 096 (with wildcard) |  | Late syphilis, latent |
|  | 097 (with wildcard) |  | Other and unspecified syphilis |
|  | 647.0 (with wildcard) |  | Syphilis complicating pregnancy childbirth or the puerperium |
| *Gonococcal Infection* | 098 (with wildcard) |  | Gonococcal infections |
|  | 647.1 (with wildcard) |  | Gonorrhea complicating pregnancy or the puerperium |

**Appendix Table 2.** Multilevel Analysis of Individual Heterogeneity and Discriminatory Accuracy (MAIHDA) analysis of HIV Testing in Medicaid data, 2012. (Not including claims for pregnancy)

| Measure of Association | Model 1 (RE only) | Model 2 (RE + FE) |
| --- | --- | --- |
| SMI |  |  |
| No SMI |  | ***Reference*** |
| SMI |  | 1.19 (1.01, 1.39) |
| Sex |  |  |
| Female |  | ***Reference*** |
| Male |  | 0.57 (0.49, 0.67) |
| Race |  |  |
| White |  | ***Reference*** |
| Black |  | 2.16 (1.70, 2.75) |
| Hispanic |  | 2.32 (1.82, 2.95) |
| API |  | 1.04 (0.82, 1.34) |
| American Indian |  | 0.93 (0.71, 1.22) |
| Age Group |  |  |
| 15-29 |  | ***Reference*** |
| 30-49 |  | 0.67 (0.55, 0.81) |
| 50-64 |  | 0.40 (0.33, 0.48) |
| Measures of variance and discriminatory accuracy | | |
| Variance | 0.691 | 0.299 |
| VPC | 0.127 | 0.026 |
| PCV | - | 0.567 |
| AUROC | 0.681 | 0.681 |

**Appendix Table 3.** Multilevel Analysis of Individual Heterogeneity and Discriminatory Accuracy (MAIHDA) analysis of Retention in HIV Care (≥2 CD4 or Viral Load tests ≥90 days apart) in Medicaid data, 2012. (Not including claims for pregnancy)

| Measure of Association | Model 1 (RE only) | Model 2 (RE + FE) |
| --- | --- | --- |
| SMI |  |  |
| No SMI |  | ***Reference*** |
| SMI |  | 1.28 (1.11, 1.48) |
| Sex |  |  |
| Female |  | 0.81 (0.70, 0.94) |
| Male |  | ***Reference*** |
| Race |  |  |
| White |  | ***Reference*** |
| Black |  | 1.08 (0.91, 1.30) |
| Hispanic |  | 0.97 (0.80, 1.17) |
| API |  | 0.51 (0.33, 0.76) |
| American Indian |  | 1.22 (0.76, 1.95) |
| Age Group |  |  |
| 15-29 |  | ***Reference*** |
| 30-49 |  | 2.20 (1.77, 2.72) |
| 50-64 |  | 2.80 (2.27, 3.46) |
| Measures of variance and discriminatory accuracy | | |
| Variance | 0.513 | 0.173 |
| VPC | 0.074 | 0.009 |
| PCV | - | 0.663 |
| AUROC | 0.579 | 0.578 |

**Appendix Table 4.** Multilevel Analysis of Individual Heterogeneity and Discriminatory Accuracy (MAIHDA) analysis of HIV Testing in Medicaid data including substance use disorder and sexually transmitted infections as covariates, 2012.

| Measure of Association | Model 1 (RE only) | Model 2 (RE + FE) |
| --- | --- | --- |
| SMI |  |  |
| No SMI |  | ***Reference*** |
| SMI |  | 1.11 (0.99, 1.23) |
| Sex |  |  |
| Female |  | ***Reference*** |
| Male |  | 0.73 (0.66, 0.82) |
| Race |  |  |
| White |  | ***Reference*** |
| Black |  | 2.14 (1.82, 2.51) |
| Hispanic |  | 2.39 (2.01, 2.80) |
| API |  | 1.14 (0.96, 1.35) |
| American Indian |  | 0.89 (0.73, 1.08) |
| Age Group |  |  |
| 15-29 |  | ***Reference*** |
| 30-49 |  | 0.78 (0.68, 0.89) |
| 50-64 |  | 0.53 (0.46, 0.61) |
| High Risk Pregnancy |  | 1.92 (1.80, 2.03) |
| Normal Risk Pregnancy |  | 7.32 (7.10, 7.61) |
| Substance Use Disorder |  | 2.32 (2.27, 2.39) |
| STIs |  | 5.16 (4.95, 5.42) |
| Measures of variance and discriminatory accuracy | | |
| Variance | 0.691 | 0.203 |
| VPC | 0.127 | 0.012 |
| PCV | - | 0.707 |
| AUROC | 0.681 | 0.731 |

RE = Random Effects; FE = Fixed Effects; SMI=Severe Mental Illness; API=Asian and Pacific Islander; VPC=Variance Partition Coefficient; PCV=Proportional Change in Variance; AUROC=Area Under the Receiver Operator Curve

**Appendix Table 5.** Multilevel Analysis of Individual Heterogeneity and Discriminatory Accuracy (MAIHDA) analysis of Retention in HIV Care (≥2 CD4 or Viral Load tests ≥90 days apart) in Medicaid data including substance use disorder and sexually transmitted infections as covariates, 2012.

| Measure of Association | Model 1 (RE only) | Model 2 (RE + FE) |
| --- | --- | --- |
| SMI |  |  |
| No SMI |  | ***Reference*** |
| SMI |  | 1.28 (1.12, 1.48) |
| Sex |  |  |
| Female |  | 0.83 (0.72, 0.95) |
| Male |  | ***Reference*** |
| Race |  |  |
| White |  | ***Reference*** |
| Black |  | 1.07 (0.91, 1.27) |
| Hispanic |  | 0.98 (0.82, 1.17) |
| API |  | 0.49 (0.33, -0.74) |
| American Indian |  | 1.22 (0.76, 1.95) |
| Age Group |  |  |
| 15-29 |  | ***Reference*** |
| 30-49 |  | 2.16 (1.75, 2.66) |
| 50-64 |  | 2.77 (2.25, 3.39) |
| High Risk Pregnancy |  | 1.80 (1.11, 2.94) |
| Normal Risk Pregnancy |  | 0.41 (0.28, 0.63) |
| Substance Use Disorder |  | 0.90 (0.84, 0.96) |
| STIs |  | 1.36 (1.22, 1.52) |
| Measures of variance and discriminatory accuracy | | |
| Variance | 0.513 | 0.16 |
| VPC | 0.074 | 0.008 |
| PCV | - | 0.688 |
| AUROC | 0.579 | 0.583 |

RE = Random Effects; FE = Fixed Effects; SMI=Severe Mental Illness; API=Asian and Pacific Islander; STIs= Sexually Transmitted Infections; VPC=Variance Partition Coefficient; PCV=Proportional Change in Variance; AUROC=Area Under the Receiver Operator Curve

**Appendix Figure 1.** Predicted mean probability of HIV testing in 2012 Medicaid population by intersectional positions (race/ethnicity, sex, age, mental illness). (Not including claims for pregnancy)


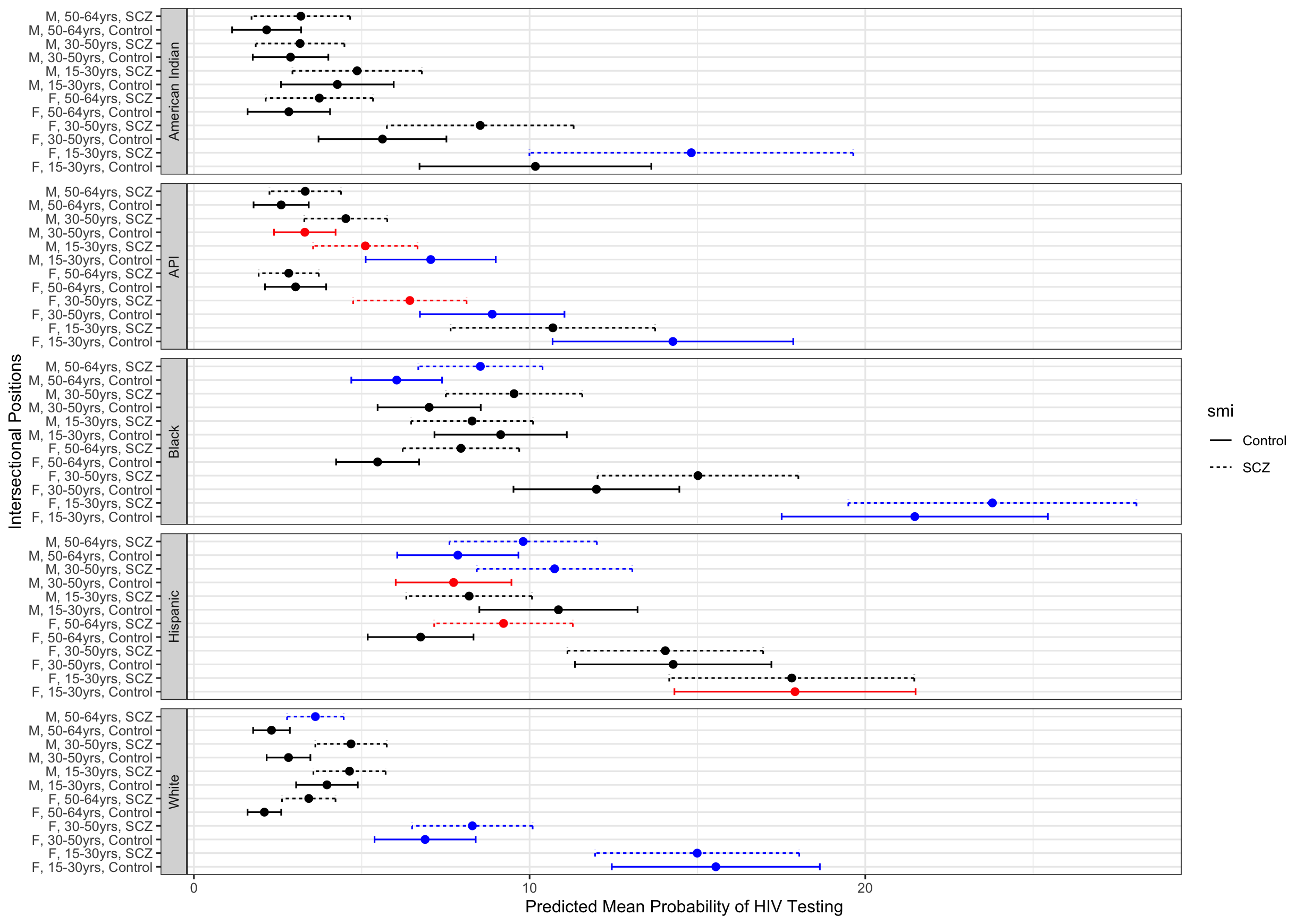


**Black dot** and error bar indicates that predicted mean prevalence is as expected based on additive combination of fixed effects. **Red dot** and error bar indicates that predicted mean prevalence is less than expected based on additive combination of fixed effects (antagonistic interaction). **Blue dot** and error bar indicates that predicted mean prevalence is more than expected based on additive combination of fixed effects (synergistic interaction).

**Appendix Figure 2.** Predicted mean probability of retention in HIV care in 2012 Medicaid population by intersectional positions (race/ethnicity, sex, age, mental illness). (Not including claims for pregnancy)


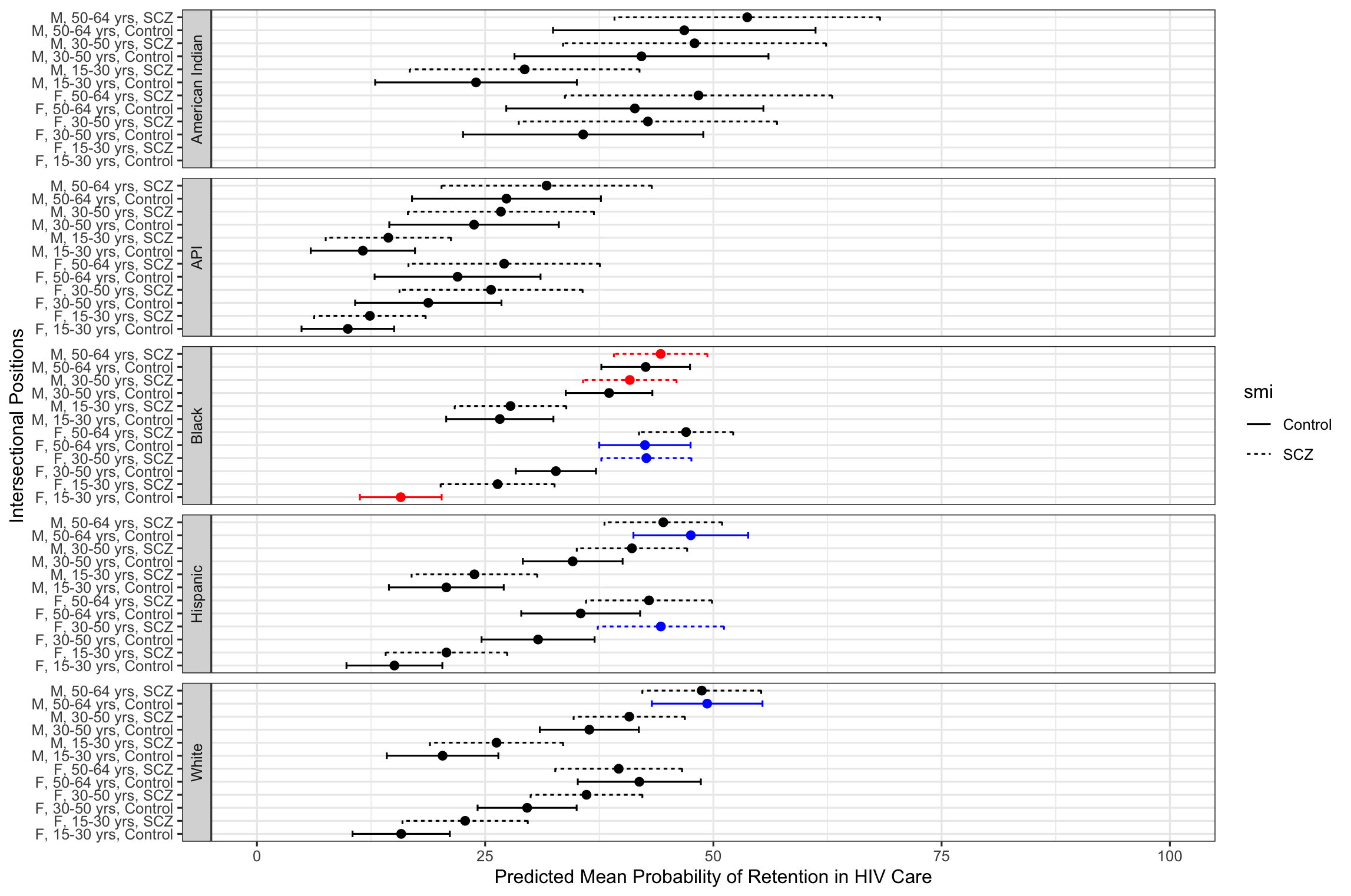


**Black do**t and error bar indicates that predicted mean prevalence is as expected based on additive combination of fixed effects. **Red dot** and error bar indicates that predicted mean prevalence is less than expected based on additive combination of fixed effects (antagonistic interaction). **Blue dot** and error bar indicates that predicted mean prevalence is more than expected based on additive combination of fixed effects (synergistic interaction).

**Appendix Figure 3.** Predicted mean probability of HIV testing in 2012 Medicaid population by intersectional positions (race/ethnicity, sex, age, mental illness). (including pregnancy, substance use disorder, and sexually transmitted infections as additional fixed effects.)


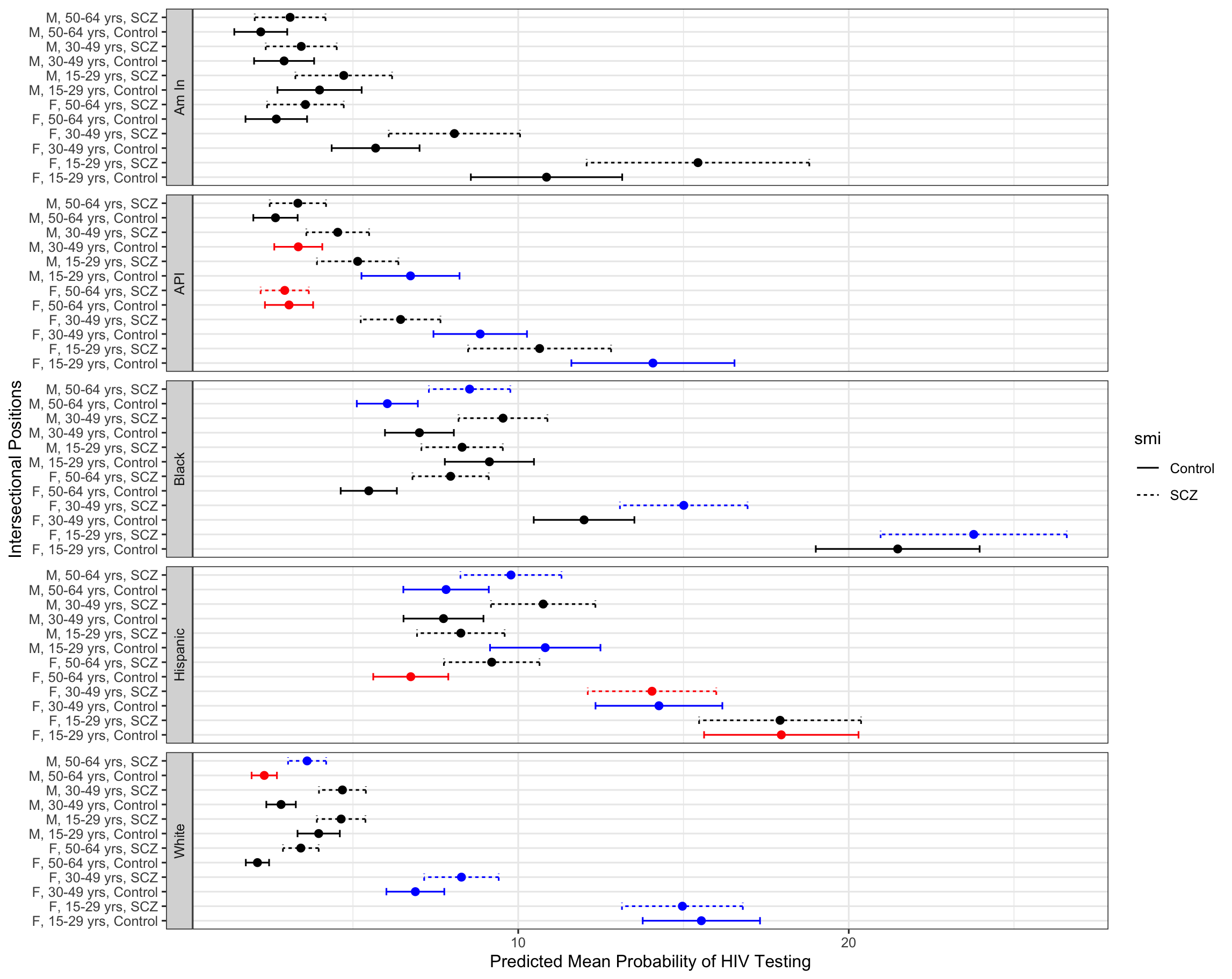


**Black dot** and error bar indicates that predicted mean prevalence is as expected based on additive combination of fixed effects. **Red dot** and error bar indicates that predicted mean prevalence is less than expected based on additive combination of fixed effects (antagonistic interaction). **Blue dot** and error bar indicates that predicted mean prevalence is more than expected based on additive combination of fixed effects (synergistic interaction).

**Appendix Figure 4.** Predicted mean probability of retention in HIV care in 2012 Medicaid population by intersectional positions (race/ethnicity, sex, age, mental illness). (including pregnancy, substance use disorder, and sexually transmitted infections as additional fixed effects.)


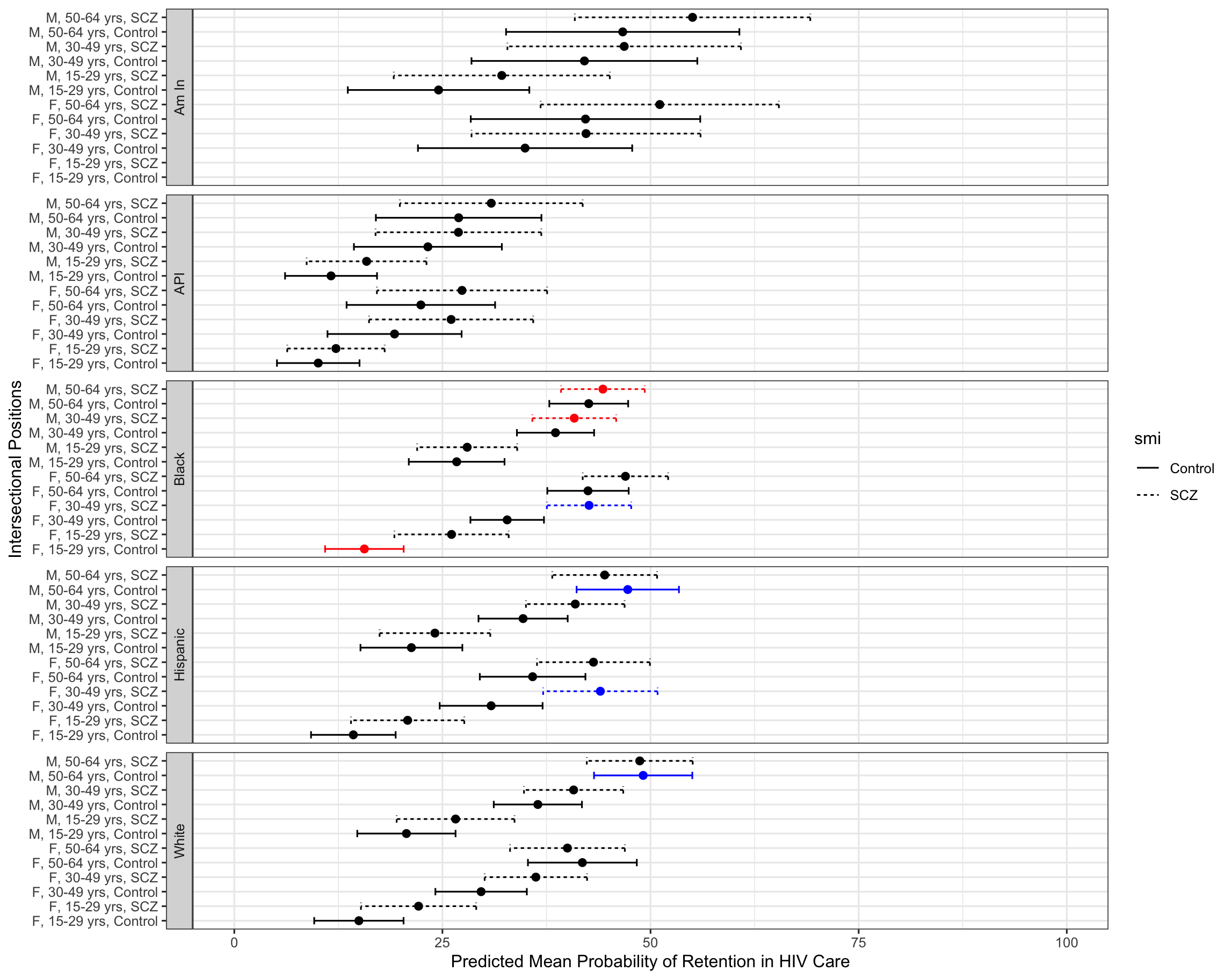


**Black do**t and error bar indicates that predicted mean prevalence is as expected based on additive combination of fixed effects. **Red dot** and error bar indicates that predicted mean prevalence is less than expected based on additive combination of fixed effects (antagonistic interaction). **Blue dot** and error bar indicates that predicted mean prevalence is more than expected based on additive combination of fixed effects (synergistic interaction).
